# Analysis of viromes and microbiomes from pig fecal samples reveals that phages and prophages rarely carry antibiotic resistance genes

**DOI:** 10.1038/s43705-021-00054-8

**Published:** 2021-10-25

**Authors:** Maud Billaud, Quentin Lamy-Besnier, Julien Lossouarn, Elisabeth Moncaut, Moira B. Dion, Sylvain Moineau, Fatoumata Traoré, Emmanuelle Le Chatelier, Catherine Denis, Jordi Estelle, Caroline Achard, Olivier Zemb, Marie-Agnès Petit

**Affiliations:** 1 Université Paris- Saclay, INRAE, AgroParisTech, Micalis Institute, Jouy-en-Josas, France; 2 Pherecydes Pharma 22 Bd Benoni Goullin, Nantes, France; 3 Département de biochimie, de microbiologie et de bio-informatique, Faculté des sciences et de génie, Université Laval, Quebec City, QC G1V 0A6 Canada; 4 Groupe de recherche en écologie buccale, Faculté de médecine dentaire, Université Laval, Quebec City, QC G1V 0A6 Canada; 5 Felix D’Hérelle Reference Center for Bacterial Viruses, Université Laval, Quebec City, QC G1V 0A6 Canada; 6 Université Paris-Saclay, INRAE, MGP, Jouy en Josas, France; 7 Université Paris-Saclay, INRAE, GABI, Jouy-en-Josas, France; 8 GenPhySE, Université de Toulouse, INRAE, ENVT, F-31326 Castanet-Tolosan, France

**Keywords:** Bacteriophages, Microbiome

## Abstract

Understanding the transmission of antibiotic resistance genes (ARGs) is critical for human health. For this, it is necessary to identify which type of mobile genetic elements is able to spread them from animal reservoirs into human pathogens. Previous research suggests that in pig feces, ARGs may be encoded by bacteriophages. However, convincing proof for phage-encoded ARGs in pig viromes is still lacking, because of bacterial DNA contaminating issues. We collected 14 pig fecal samples and performed deep sequencing on both highly purified viral fractions and total microbiota, in order to investigate phage and prophage-encoded ARGs. We show that ARGs are absent from the genomes of active, virion-forming phages (below 0.02% of viral contigs from viromes), but present in three prophages, representing 0.02% of the viral contigs identified in the microbial dataset. However, the corresponding phages were not detected in the viromes, and their genetic maps suggest they might be defective. We conclude that among pig fecal samples, phages and prophages rarely carry ARG. Furthermore, our dataset allows for the first time a comprehensive view of the interplay between prophages and viral particles, and uncovers two large clades, inoviruses and Oengus-like phages.

## Introduction

The spread of antibiotic resistance genes (ARGs) is a major health concern, and powerful sequencing techniques are now making it possible to examine the contribution of mobile genetic elements to their spread. The main mobile vectors currently recognized as responsible for spreading ARGs are conjugative plasmids and integrative conjugative elements (ICEs, also known as conjugative transposons) [1]. In both cases, ARG spread occurs through conjugation, a process that involves the formation of a conjugative pilus, the contact between a donor and a recipient bacterium, and the transfer of the genetic element. ARG are frequently found in pig fecal samples [2], and their associated mobile genetic elements start to be well documented. For instance, analyses were conducted on a set of metagenomics contigs that were cloned on bacterial artificial chromosomes. Eleven of them were conferring resistance to tetracycline to *Escherichia coli*, and nine of the *tet* genes were present on mobile genetic elements (plasmids or ICEs) [3].

Interestingly, bacteriophages, another category of mobile genetic elements, apparently rarely encode ARGs [4]. Phages can usually be divided into two large groups based on their lifestyle. Virulent phages inject their genetic material into bacteria, replicate and lyse their host cells, thereby releasing several new virions at the end of this lytic cycle. Temperate phages are able to alternate between the lytic cycle and a dormant stage where they maintain their genetic material within the bacterial genome as prophages (following an integration step or as plasmids). While dormant, prophages may still express a few of their genes, including some which are beneficial to their host, such as *bor*, which is involved in resistance to serum complement killing [5], and the iron transporter *sitABCD* genes [6]. In theory, a temperate phage and its expression profile in a prophage state would offer an efficient process for the dissemination of ARGs. So, why have they so rarely been detected?

Initially, their absence was noted on the basis of single phage biology methods, such as phage culturing and sequencing, and therefore the investigations lacked breadth. On the other hand, qPCR studies have reported the presence of ARGs in virus-enriched environmental samples from natural waters [7], wastewater plants [8], or human/animal fecal samples [9, 10]. However, virome preparations are often highly contaminated by bacterial DNA [11, 12], making it difficult to discern whether the ARG originates from viral or bacterial DNA using qPCR alone. A distinct process through which phages can mobilize ARG is generalized transduction. During this process, instead of packaging their own genome in their capsid, some phage particles will package bacterial DNA (sometimes at high frequencies [13]). This process is far from universal in phages, and the fraction of phages capable of performing such task is unknown.

Shotgun metagenomics studies of enriched viral fractions (viromes) and improved programs for assembling short reads into quality contigs can now help determine whether ARGs are found in phage genomes. ARGs were recently investigated in the virome from wastewater samples, and they were found to be scarce compared to other genetic elements [14, 15]. However, several reports have raised a concern with pig fecal samples, which appeared to be rich in phage encoded ARG [9, 16, 17].

Another approach for investigating the ARG content of phages is to study prophage regions of completely assembled bacterial genomes. Recent reports on pathogenic bacterial species such as *Acinetobacter baumanii* or *Streptococcus suis* found prophage regions with ARGs [18–20]. In these in silico studies, the difficulty lies in the ability to discern whether such prophages are defective or still functional and able to, for instance, complete lytic cycles, form viral particles, and therefore spread ARG in bacterial populations.

Here, with respect to the ARG content in pig fecal viromes, we elected to extract and analyse new samples, because the presence of bacterial DNA contamination was either clearly mentioned in previous reports [17], or it was poorly documented, by the absence of detection of a DNA band after PCR with 16 S universal primers. Moreover, we sequenced in parallel the purified virome and the total microbiota of the same samples to have access to the prophage content and be able to test whether they were active (i.e., present also in the virome fraction). Our study presents the results of these analyses performed using 14 pig fecal samples collected from farms with varying degrees of antibiotic usage and reports the presence and absence of ARG among microbiota and viral particles, respectively.

## Material and methods

### Sample preparation sequencing and assembly

Starting from 14 pig or piglet fecal samples, parallel DNA extractions were conducted for the purified viral fraction, named VP, and the total microbiota, named P, using standard procedures. All details are available in the Supplemental Information. Raw sequencing data and SPAdes assembly outputs (>2 kb, contigs for VP samples, scaffolds for P,134 before the clustering step at 95% identity) are accessible through the NCBI, Bioproject PRJNA741980.

### Analysis and labeling of the bona fide viral contigs within the VP dataset

The analysis of all VP contigs of a size above 2 kb was performed in two steps. From the 7755 VP contigs, the 2480 with the highest RPKM (above 100) were first analyzed. Several programs and methods were applied to identify non viral contigs (bacteria and plasmids, see Supplementary Fig. 1). Virsorter [21] recognized 23% (568 contigs) of these contigs as viral (all categories were accepted, cat1 and cat2 were vastly dominant). The Inovirus detector [22] was then run on the remaining contigs and 143 inoviruses (putative or confirmed, most were circular). To add contigs corresponding to phages recently added into public databases, MegaBLAST was run against the nt database of NCBI (April 2020, E-val below 10^−16^). As bacterial hits could also correspond to prophages, all BLASTn hits, as well as the remaining unclassified contigs were taken for a search centered on proteins, making use of PHROG, a database of phage proteins [23] (see https://phrogs.lmge.uca.fr). For this, an HMMER hmmscan [24] of proteins from all remaining contigs was run against PHROG profiles, and all contigs with at least 5 orfs and 35% of them homologous to a PHROG family (E-value below 10^-12^) were counted as phages. In total, this BLASTn + PHROG step allowed for the recognition of 966 additional contigs from the following origins: 654 phage, 232 eukaryotic viral (collectively designated below as “viruses” for short, while “phages” is kept for bacterial viruses), 72 bacterial and 8 plasmid fragments. Next, the VIBRANT classifier [25] was used on the remaining uncharacterized contigs, adding 325 phages and 29 viruses. In the end, 1951 contigs were found to be of viral origin, 72 bacterial, 8 plasmidic, and only 449 contigs of the 2480 (18%) remained uncharacterized.

In a parallel study on infant gut viromes, various tools dedicated to the recognition of viral contigs were compared, and VIBRANT was among the best [26]. The 1951 viral contigs detected in the top 2480 contigs with high abundance (as described above, see also Supplementary Fig. 1) were therefore verified using VIBRANT. A high degree of overlap was observed between the pre-VIBRANT sorting and VIBRANT detection for all *Caudovirales* and *Petitvirales* genomes (92.5% and 99% were detected by VIBRANT, respectively). VIBRANT was also successful at detecting eukaryotic viruses, as 79% of them were detected. VIBRANT was less successful at detecting inoviruses (35% detected).

In a second step, we therefore used VIBRANT and the Inovirus detector on the remaining 5275 low-abundance contigs and obtained an additional 3654 viral and 201 inovirus contigs. In total, 5806 VP contigs (75%) were recognized as viral (and 77.3% of the contigs treated by VIBRANT, which filters for contigs with ≥2 kb and 4 ORFs). Of these 5806 viral contigs, 44% were considered complete or high quality by CheckV [27].

### Other bio-informatic methods

ARG search, VP viral contig classification, host prediction for the viral contigs, as well as the procedure to partition active phage versus inactive prophage contigs within the microbial P dataset, are all described in the Supplementary Information.

## Results

### Among most abundant virome contigs, only 18% correspond to unclassified contigs

Viral fractions of the 14 pig fecal samples were purified, separated on gradients, their ss- and ds-DNA extracted, amplified by multiple displacement amplification (MDA), and sequenced, as described in Supplementary Information. Reads were assembled into contigs, those of a size above 2 kb were retained, and clustered at the species level (>95% nt identity). Viral reads were mapped back onto the 7755 contigs obtained, and relative abundance of these contigs were computed to generate the so-called “VP” (for “virome-de-porc”) matrix.

The 2480 contigs with total RPKM values above 100 were then selected for in-depth viral sequence identification (see “Methods” and Supplementary Fig. 2A). Overall, 82% of all abundant contigs (2031) could be classified either as viral or, in a few cases, bacterial or plasmidic, therefore reducing unclassified contigs (the so-called “viral dark matter”) to only 18% of the sub-set of abundant VP contigs (Supplementary Fig. 2B). Among these classified contigs, 83% were phages, and 13% eukaryotic viruses (Supplementary Fig. 2C), and the remaining contigs were bacterial or plasmid DNA. Most phages belonged to the *Petitvirales* order (52% of phages), which is consistent with previous research [28], and the most common viruses were from the *Smacoviridae* family (31 % of viruses, Supplementary Fig. 2D).

**Fig. 1:**
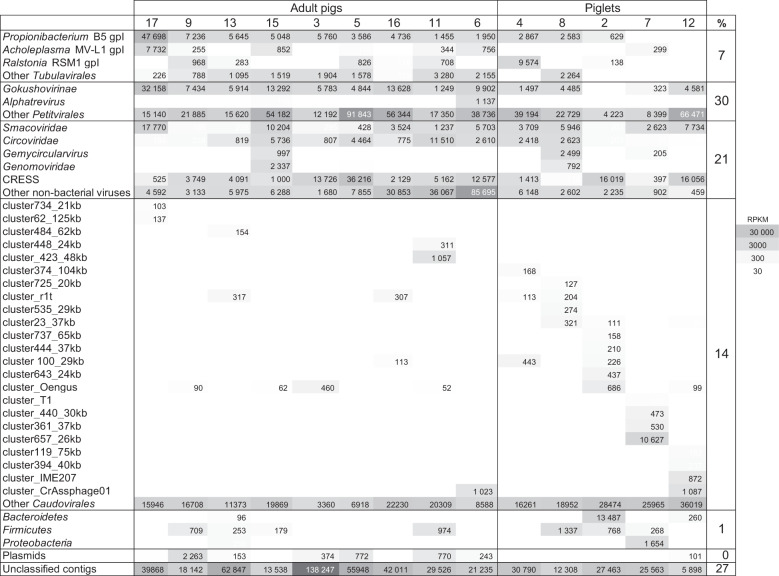
Abundance matrix of the 2480 most abundant virome contigs, grouped into clades. Abundances are expressed as RPKM (gray scale on the right). The last column shows the percentage of total RPKM in each contig category: *Tubulavirales*, *Petitvirales*, non-bacterial viruses, *Caudovirales*, Bacteria, Plasmids, and Unclassified.

An overview of the abundance matrix of these 2480 most abundant contigs, grouped by clades, is shown Fig. 1 (see full matrix in Supplementary Table 1). It should be noted that ssDNA viruses abundances cannot be compared to dsDNA ones (*Caudovirales*), due to their over-amplification during the MDA step [29, 30]. Still, *Petitvirales* represented 30% of total abundance, followed by non-bacterial viruses (21%) and *Tubulavirales* (7%). *Tubulavirales* contigs (described in detail below) were grouped according to their type of morphogenesis protein, encoded by *gpI*. We noted a gradient of abundance in *Tubulavirales*, with adults containing most of them (samples are organized in the matrix following this gradient). Among *Caudovirales*, some of the contigs were grouped into higher order taxons (see below). In these cases, the contigs were labeled with the name of a similar reference phage genome when available, and were otherwise assigned a vCONTACT2 [31] cluster number (followed by the size of the circular or largest contig). Still, most signals came from unclustered contigs. We noted that piglets had significantly higher abundances of *Caudovirales* compared to adults (*P* = 0.0036, Student t-test), and fewer ssDNA eukaryotic viruses (not significant, *P* = 0.07). The remaining lower abundance contigs were also classified,and 72% were found to be viral. We conclude that the majority of contigs from these highly purified viromes were indeed viral in nature, with a substantial proportion being ssDNA viruses.

### The few antibiotic resistance genes found in viromes are not encoded by phages

ARG were then searched among the complete set of 8090 VP contigs of a size above 2 kb (before clustering, and regardless of their abundance) in viromes. Contigs that were not predicted to be viral were also included (Table 1, see Supplementary Information). The search against ResFam profiles [32] revealed 8 VP contigs encoding 10 putative ARG, 5 of which were also retrieved with a BLASTp search against MUSTARD [33]. The 5 missing ARG encoded ABC transporters, a category which is simply not included in MUSTARD. Finally, a more stringent search of confirmed ARG with Resfinder [34] reported only 3 of the 10 initially uncovered ones (Table 1). Interestingly, in one of the 8 ARG-positive contigs (VP7_NODE302), Resfinder identified a *tet(40)* gene undetected with the two previous tools.

**Table 1:** The eight ARG-positive contigs assembled from viromes.

NODE	ARG name ResFam	ResFam (E-val)	Resfinder	CONTIG properties			
				phage	best BLASTn hit	size, nt (©=circular)	RPKM
				VIBRANT			in viromes
VP8_NODE183	ABC_efflux	2.9e–97	NO	No	*Parabacter. distasonis*	16 427	15
VP2_NODE298	MexH	2.2e–42	NO	No	Alistipes	13 316	7
	RND_efflux	0	NO				
VP7_NODE302	tet(40)	NO	YES	No	*Eubacterium maltosivorans*	7 945	11.9
	ANT9	1.3e–80	NO				
VP7_NODE1	ABC_efflux	7.9e–89	NO	No	Clostridiales	301 151	35.9
VP7_NODE2	ABC_efflux	5.1e–84	NO	No	–	230 004	37.9
VP7_NODE3	tet(W)	0	YES	No	–	226 564	33.4
VP8_NODE804	TEM	1.8e–205	YES	No	*E. coli* cloning vector pET11a	5 676 ©	32.1
	tet_MFS_efflux	1.9e–80	NO				
VP12_NODE394	APH3'	3.2e–113	YES	Yes	*C. difficile* plasmid pCd13	5 473 ©	57.7

Detailed inspection of the 8 contigs revealed that none of them originated from phages, despite a positive VIBRANT [25] prediction for one of them (contig VP12_NODE394, last line in Table 1). This phage candidate, however, turned out to be 99.8% identical to plasmid pCd13 of *Clostridioides difficile* (Accession MH229772). In addition, this 5.4 kb contig was circular and contained a 1.5 kb deletion compared to pCd13. We therefore conclude that it is rather a plasmid than a phage. Of note, the second circular contig, VP8_NODE804, was 100% identical to synthetic plasmid pET11a with its *Nde*I restriction site mutated. We think this laboratory vector comes from the control DNA of the Genomiphi kit used to prepare DNA prior sequencing, suggesting that the sequencing depth was sufficient to detect even minor contaminants. Homologs for all remaining, non phage contigs were searched with BLASTn against NCBI nt database (May 2019) and most of them had a taxonomic association that is typical of intestinal bacterial species (Table 1).

We noted that the six ARG-positive contigs of chromosomal origin came from the viromes with less viral enrichment (Supplementary Table 2). Given the lower level of purity, it seems likely that these fragments came from remaining free bacterial DNA rather than viral particles. In contrast, the plasmid contig homologous to pCd13 from *Clostridioides difficile* came from a highly purified virome, making bacterial contamination unlikely. This plasmid may have been carried over by generalized transduction.

Taken altogether, the few ARG-positive contigs we found in highly purified pig viromes were not phage-encoded, even in samples originating from farms with heavy antibiotic use, such as sample 16. Compared to the 5806 contigs positively recognized as viral, this places the frequency of ARG-encoded viral contigs below 0.02%. The maximal ARG gene ratio over total viral orfs was below 1.2 10^−5^, or below 0.015 per Mb of assembled viral contigs.

### Pig viromes are rich in inoviruses and Oengus-like phages

**Fig. 2:**
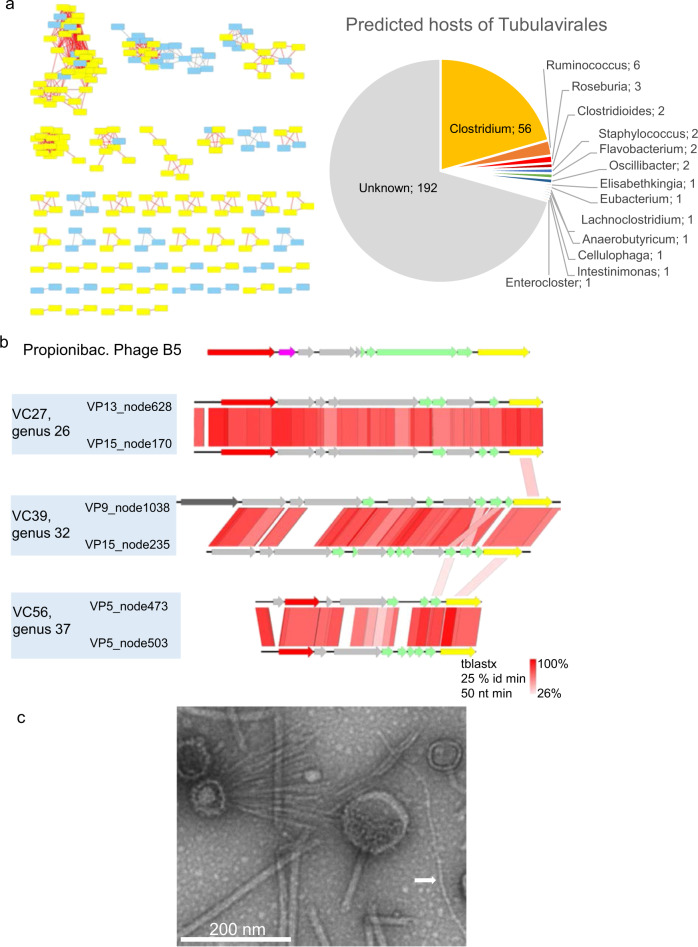
Properties of circular pig inoviruses. **a**, left VContact2 map of all complete genomes that could be clustered. Those encoding a *gpI* homologous to the one of Propionibacterium phage B5 are highlighted in yellow; **a**, right, host predicted for all circular genomes. **b** Genome alignments (tBLASTx) and Easyfig map of several inoviruses encoding a B5-like *gpI* gene. Yellow: gene encoding morphogenesis protein I, red: *rep* gene for replication initiation, green: structural genes (with transmembrane domains), pink: *ssb* (single-strand DNA binding) gene. Gray: gene of unknown function. Dark gray: putative phosphoadenosine phosphosulfate reductase. **c** Transmission electron microscope image of virome VP17, the white arrows point to a thin filament that may correspond to an inovirus (length> 420 nm, width, 8 nm).

Following the examination of ARGs, we explored the main characteristics of these virome assemblies. Among the rich viral content, two main clades emerged. First, 344 VP contigs (272 of them circular) were found to correspond to inoviruses—filamentous phages performing chronic bacterial infections [35]. Taxonomically, the best characterized family of inoviruses, *Inoviridae*, is now included into a *Tubulavirales* order and the richness of this order has recently been put to light [36]. Most of inovirus contigs (181 of the 344, 52%) possessed the *gpI* type of Propionibacterium phage B5 (Fig. 1), the only known filamentous phage infecting a Gram-positive host [37]. Adult pig viromes were particularly rich in this group, compared to piglets. The 272 complete circular genomes were taken for a deeper analysis (Fig. 2, details about all circular genomes are reported in Suppl. Table 3). Their clustering using vCONTACT2 [31] indicated that they were very diverse, forming 50 clusters of a quality above 0.5, and leaving aside 93 singletons. None of the clusters included any of the 10 reference inoviruses available, not even phage B5. The 118 circular and clustered genomes with a B5 *gpI* are highlighted in yellow on the vCONTACT2 map (Fig. 2a).

We tried to predict the bacterial hosts of these inoviruses with circular genomes. A search of nearly-identical CRISPR spacers led to the prediction of four possible *Firmicute* hosts: two *Roseburia*, a *Ruminococcus* and a *Lachnoclostridium*. A complementary search using WiSH [38] or BLASTn added 67 predictions, most often in the *Clostridium* genus (Fig. 2a, right). Consistent with these predictions, a filamentous phage infecting *Clostridium acetobutylicum* NCIB6444 was isolated (but its genome not sequenced) 30 years ago [39].

Pairwise tBLASTx genome comparisons between B5 and some of the VP genomes with a similar *gpI* revealed similar genetic organizations, although the B5-encoded *gpI* was too distantly related to those of pig genomes to appear in the results (Fig. 2b). Finally, we noticed that electron transmission microscopy images of the VP17 virome, which contained the most inoviruses, revealed some long filaments that are typical of inoviruses (Fig. 2c). We conclude that novel, *Firmicute*-infecting inoviruses are present in pig fecal samples.

The second notable characteristic of these pig viromes was identified by performing a vCONTACT2 clustering analysis [31] of the 2991 *Caudovirales* VP contigs, together with 2214 reference phage genomes (see Supplementary Information). Among the seven most populated clusters (listed in Suppl. Table 4), there was a particularly large one of 98 elements, 94 of which originated from pig virome contigs. Of note, four reference phage genomes were connected to them. Three of these four phages are virulent and infect *Actinobacteria*, including the Rhodococcus phages ReqiPoco6 and ReqiPepy6 [40], and Arthrobacter phage Mudcat (accession NC_031224). The fourth is the temperate phage Oengus, which infects the *Firmicute* *Faecalibacterium prausnitzii* [41].

**Fig. 3:**
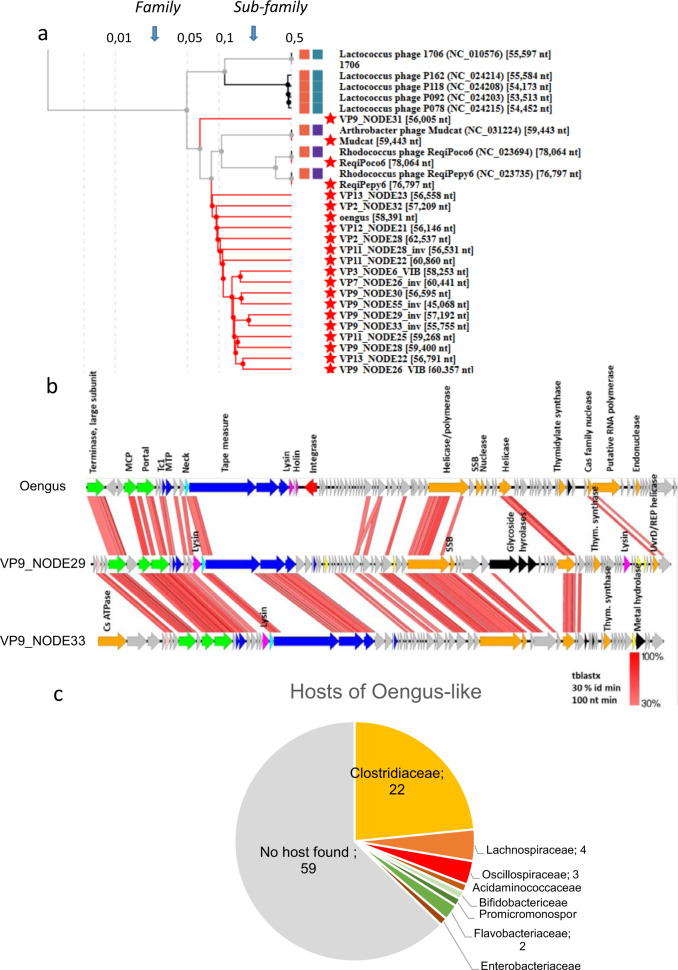
Characteristics of the Oengus cluster. **a** Tree representation of the 17 largest Oengus-like contigs together with close reference phage neighbors in the global VipTree [42], which builds a distance based on shared genes, starting from nt sequences and tBLASTx comparisons. Red stars indicate all genomes that were added to the reference VipTree. **b** Whole genome alignments (Easyfig map, with tBLASTx option) comparing two relatively close VP nodes (**a**) and Oengus. MCP: major capsid protein, Tc1: Tail completion 1, SSB: single-strand binding protein. **c** Predictions of infecting hosts for the 94 contigs of the Oengus-like cluster.

The 17 largest genomes of this cluster (45–62 kb) were then compared to already classified phage genomes with ViPTree [42] (Fig. 3a). The ViPTree branch lengths were used to delimit family boundaries (branch lengths extending into the 0.01–0.05 region), and subfamily boundaries (between 0.1 and 0.5). Members of the large vCONTACT2 cluster were connected by deep branches (0.1), suggesting the clade includes more than one viral genus. Furthermore, phage Oengus was closest to all but one of these VP contigs. Surprisingly, among the 38 nearly complete genomes (>40 kb), only three contained an integrase, suggesting a clade of mostly virulent lifestyle. Alignments of Oengus with two complete genomes of this clade showed that only a few genes were shared, confirming further that this cluster represents a taxon beyond the genus level (Fig. 3b). Finally, most frequently predicted hosts belonged to the *Clostridiaceae* family (Fig. 3c). In addition, for two of them, a few matching CRISPR spacers were found in strains of *Anaeromassilibacillus*, a genus that belongs to the same *Oscillospiraceae* family as *F. prausnitzii*, the host of phage Oengus. We recently proposed the family name Sisseviridae for this phage clade (extending up to Lactococcus phage 1706, Fig. 3a), which currently comprises 72 genera and 254 species [27].

### Pig microbiota contains a high abundance of viruses in addition to six main bacterial phyla

We next investigated whether ARGs could be detected on prophages that are embedded in bacterial genomes. For this, we extracted, sequenced and assembled the DNA from the global fecal samples, following a distinct protocol for DNA extraction (see Supplementary Information, and Supplementary Fig. 2a for the overall analysis pipeline). After assembly and dereplication, a final binning step was performed to aggregate contigs from the same microbial species, using the VAMB tool [43]. To estimate the main phyla present in the 14 pig fecal samples, a taxonomic affiliation with Kaiju was performed on the 220,000 contigs of a size above 2 kb.

**Fig. 4:**
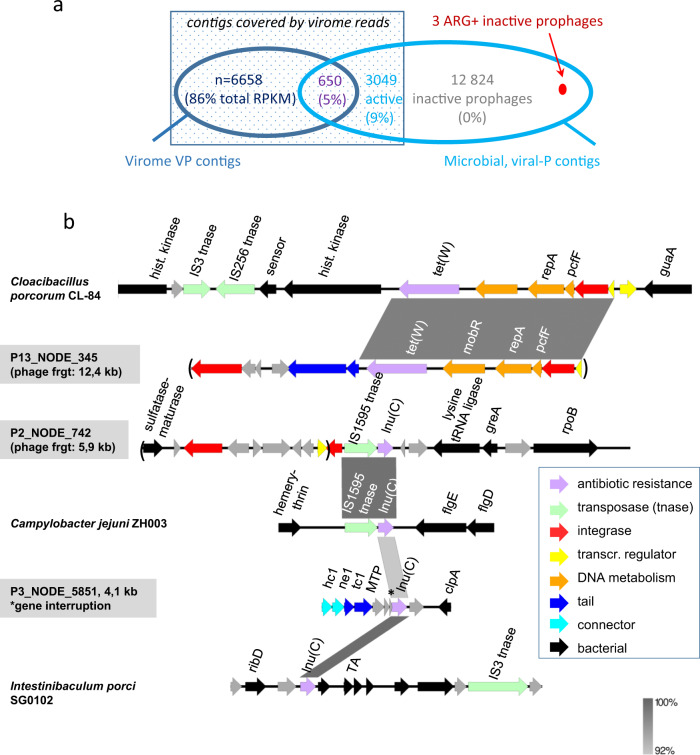
Overview of the ARG found in pig phages and prophages contigs. **a** Overlap between viral contigs assembled from virome fractions (VP) and those recognized as viral within microbiota assemblies (viral-P). Among 23 181 dereplicated viral contigs (see Supplementary Information for details), 650 were present in both contig sets, while 6658 were present exclusively in viromes. No ARG was found in these two first subsets. Among the viral-P contigs, we distinguish those “covered” by viromes reads, and therefore considered active phages (square of dark blue shade, abundance in viromes indicated as % of RPKM among the 3 subsets of contigs covered by reads), from those that correspond to dormant prophages The 3 ARG positive viral contigs (indicated in red) are among dormant prophages. **b** Genetic maps of these three prophage regions. Easyfig [53] maps (BLASTn) of shared regions with other mobile elements are shown. Phage hallmark genes (tail and connector) are colored in blue. GNAT stands for GNAT family N-acetyltransferase, TA for toxin-antitoxin system, GGDEF for GGDEF domain-containing protein.

In addition, we searched systematically for viral contigs among this microbial contig collection, using a combination of 3 criteria: (i) positive viral result according to VIBRANT [25], (ii) homologous to a viral VP contig according to BLASTn results, (iii) viral affiliation by Kaiju (see details in Supplementary Information). Since huge phages are often found in pig microbiota, we searched for them in our dataset, and found one distant homolog in the P set, and three additional among the virome VP contigs (Section 2 of Supplementary Information). Of the 220 000 contigs, 16 940 (7.7 %) were deemed of viral origin (labeled viral-P contigs below). We reasoned however, that some of these contigs might correspond to uninduced prophages and some may even be defective prophages. We therefore used the collection of virome reads to determine which of the viral-P contigs were forming viral particles (see Supplementary Information). Among these 16 940 viral-P contigs, 23.7% were covered by a significant amount of virome reads and classified as “active phages” (Fig. 4 and Supplementary Fig. 2).

**Fig. 5:**
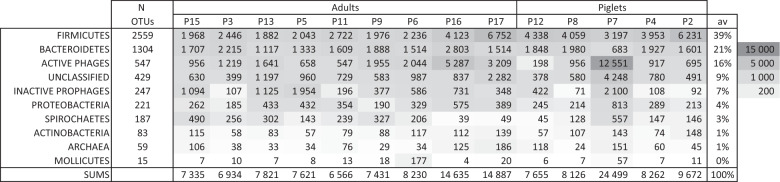
Main pig microbiota phyla. Abundances are expressed as RPKM (gray scale on the right). The average column does not include sample P7.

Figure 5 reports a synthetic representation of the main phyla detected in the pig microbiota, among OTU with abundance above 10 RPKM across the 14 samples (see details in Supplementary Table 5). In this matrix, active phages were binned and counted separately (798 contigs, binned into 547 “active phages”). The remaining viral contigs, for which no proof of activity was obtained, were either kept under their bacterial host taxonomy when available (329 contigs), or placed under “inactive prophages” otherwise.

In most samples (13/14), the three main phyla were *Firmicutes* (39%), *Bacteroidetes* (21%) and surprisingly, active phages (16%). The next microbial core phyla with abundances above 1% were *Proteobacteria*, *Spirochaetes*, *Actinobacteria* and Archaea. Aside from the active phage category, previous reports on pig microbiota described similar phyla distributions [2, 44, 45]. The *Mollicute* class was added in the matrix to highlight sample P6, which contained a particularly high level of a single *Mycoplasma* OTU, suggesting pig 6 was sick.

The last sample (piglet P7) had a distinct profile, as virus reads represented 51% of the total. More precisely, a bin of two contigs (12.6 kb total) was responsible for 52% of all viral signal. Upon closer inspection, these contigs had a typical *Salasmaviridae/Sarlesviridae* organization (Supplementary Fig. 3), but its host could not be predicted. Interestingly, the P7 sample also contained a very large 14 Mb OTU (bin of 401 contigs). A BLASTn search against the nt database of the NCBI indicated that its closest (77% identity) and most frequent (18% of contigs from this bin) parent was *Blastocystis hominis* (Eukaryote, Stramenopiles). We also noted that in this sample, richness and abundance in *Bacteroides* and *Prevotella* species was low. This suggests that there is intense predation on bacteria in the microbiota from sample P7.

### A small fraction of ARGs are found in prophages in pig microbiota

An ARG search among the 271 163 P contigs (before dereplication) retrieved 1764 ARG with ResFam and 279 with Resfinder (see Supplementary Table 6 for overall counts, and Suppl. Table 7 for the details of each contig). The large increase in ARG with ResFam is due to the inclusion of ABC transporters, which probably include false positives. We therefore concentrated on ResFinder numbers. The ResFinder counts correspond globally to 1.8 (+/−0.5) 10^−4^ ARG gene per total genes, and 0.18 (±0.05) ARG gene per Mb.

To further investigate the samples with the highest abundances of ARG-positive bacterial contigs and the category of antibiotic resistance that was prevalent, we focused on the abundance matrix of the 168 contigs that harbored the 279 ARG genes (Supplementary Table 17). Overall, a four-fold difference was observed between the most and least ARG-abundant microbiota samples (Supplementary Fig. 4a). Piglets also had higher prevalence of ARGs compared to adults (not significant, Wilcoxon-Mann Whitney test, *p* = 0.109). Contigs encoding tetracycline resistance genes were by far the most abundant, followed by aminoglycoside and macrolide resistance genes (Supplementary Fig. 4b–e), consistent with previous reports [2]. Among the *tetR*-encoding contigs, three were particularly abundant, in all samples: the most abundant one encodes a *tet(*W) gene (in P4_NODE_19877) which apparently belongs to an integrative conjugative element (ICE, see Supplementary Information). The two next most abundant contigs (*tet (40)* genes in P12_NODE_17183, 2004 bp, and P8_NODE_13797, 2606 bp) were partially overlapping and matched a plasmid reported from a study on the tetracycline resistome of bio pig farms [3]. Clearly, and as concluded also by Kazimierczak et al. [3], even though tetracycline which was used as a growth factor, is no longer used as such in Europe, the previously selected resistance genes still remain in pig microbiota. Some samples, however, and particularly for the pig from farm 6, were markedly ARG-poor. What parameter of the farm settings could explain this good record is not known at present. We conclude that globally, the microbial ARG content of these pig fecal samples was similar to that previously reported in the literature [2, 46].

We next sought whether some of the ARG-positive contigs were viral. Three were indeed viral (representing 0.02% of the 16 940 viral-P contigs), but belonged to the inactive prophages subset (Fig. 4a). These 3 contigs represent 1.7% of all 168 ARG-positive contigs, while 30 at least were present on other mobile genetic elements (plasmids and ICE, see Supplementary Table 7, 8). A genetic analysis of the 3 ARG + viral contigs revealed an ICE-prophage hybrid in one case, an IS just next to the prophage region in the second case, and a possible phage gene interruption by the ARG in the last case (star above the gene, Fig. 5b). Altogether, we conclude that our search for ARG-positive viral contigs across viromes and microbiomes retrieved none in the viromes, and 3 on inactive prophages.

## Discussion

In-depth characterization of 14 pig fecal samples revealed that ARGs are present at 0.18 genes per Mb in the total microbiota of these samples (168 contigs, 279 ARGs). However, none of the ARG-containing contigs appears to belong to active phages, since ARGs were not detected in the viral contigs assembled from viromes reads (detection limit of 0.015 ARGs per Mb of viral contigs). Assuming an average genome size of 3 Mb for bacteria, and 30 kb for phages, these ARG numbers correspond to 0.5 ARG per bacterial genome, and below 3.6 10^−4^ per phage genome. This suggests that ARG genes are at least 1000-fold less likely per viral genome, compared to bacterial genomes. We therefore conclude that even in pigs, and in samples originating from farms with heavy antibiotic usage (such as sample 16), the probability of detecting an ARG on a phage contig is at least 1000-fold lower compared to a bacterial genome.

An explanation for this contrast could be that ARG, whenever they enter prophage genomes (by transposition or recombination), tend to mutate the recipient phage, which loses then rapidly its fitness. From the bacterial point of view, one could also argue that an ARG-containing prophage is so beneficial for the host that phage domestication rates speed up in order to retain the ARG.

Indeed, comparison of the 168 ARG-positive contigs to the pool of prophages present in microbiota revealed that three of them were prophages. Among these prophages, we introduced a distinction between those corresponding to functional and active phages, producing virions and therefore covered by virome reads, and inactive prophages. A large majority of these prophages were inactive (12 824, 77% of the total pool of prophage contigs), and the three ARG-positive prophage fragments belonged to these inactive prophages (Fig. 4a). This, together with the genetic map of these fragments (Fig. 4b), suggests they are being domesticated.

In fact, the subset of ‘active phage’ contigs in total microbiota samples entailed more than prophages. DNA from virulent phages was also present, such as the dominant phage of sample P7, belonging either to *Salasmaviridae* or *Sarlesviridae* family (Supplementary Fig. 3) We can propose that the category of “active phages” also includes the “virocell” fraction, namely the fraction of phage particles replicating in bacteria at sampling time. A recent report suggests that in human microbiota, this fraction represents 25% of all viral contigs detected in total microbiota [47]. Interestingly, in our pig samples, the proportion of ‘active phage’ contigs was similar (23%). This pool of active phages constituted an important fraction of the ecosystem, as it represented in average 16% of genome abundance (RPKM) in each sample (Fig. 5). If phage burst size is around 10 in the intestine (it is estimated at 16 for the Lambda phage in the murine intestine [48]), this proportion of 16% would correspond to a maximum of 1.6% of bacteria hosting an actively replicating phage. In one case (piglet P7 sample), the active phage fraction included 50% of total RPKM. This sample hosted as well a putative Stramenopile, and its bacterial richness was strongly decreased, suggesting dysbiosis.

The comparison between viral contigs found in the virome fraction and the total microbiota is somewhat puzzling, though. Only 1097 of the 7755 virome contigs (14%) have homologs in the total microbiota. Two studies have reported similar comparisons. A limited 10% overlap has been reported by Gregory et al. [49] on 10 human fecal samples sequenced both in bulk and after viral particle filtration [50]. In a study involving 662 kid fecal samples however, the overlap was larger (30% of virome contigs overlapped microbiota viral contigs, and 50% the other way round) [51]. A technical reason for the lack of overlap between microbial and virome viral contigs is certainly phage incomplete assemblies, which may produce different fragments of the same phage in the two subsets. The fact that in the present study, some 3000 pig virome contigs, while not overlapping microbiota contigs, are nevertheless covered by microbiota reads (and reciprocally, see Supplementary Fig. 1) comforts this hypothesis. Besides this first explanation, the protocol used to extract total microbial DNA may also have extracted the DNA present in viral particles, among which ssDNA viruses would not be sequenced (indeed, 70% of the virome contigs not covered by microbiota reads corresponded to ssDNA viruses). Unless a specific step is added to bulk sequencing, virome sequencing will continue to bring more viral information relative to bulk sequencing for this reason. Finally, even if the two preceding technical problems are solved in the future, complete overlap between the two types of viral contigs will never occur, due to domesticated prophages that will never replenish the pool of active phages. A way to focus on the active temperate phages and exclude dormant prophages was recently documented [52]. Even though a complete view on the viral world is not possible upon microbiota sequencing as performed here, it offered the invaluable benefit of a glimpse at phage-bacteria interactions, and revealed the striking prevalence of phages reads (50% of total) in a dysbiotic piglet (P7).

The significant progress of all tools designed to sort out viral genomes allowed us to characterize 82% of the 2480 most abundant contigs of the 14 virome samples. Among them, two prominent phage clades were distinguished, inovirus infecting Gram-positive hosts (Fig. 2), and dsDNA phages similar to Faecalibacterium phage Oengus (Fig. 3 [41]). Even though viral samples had been purified on gradients, some bacterial and plasmid DNA was still present. We could not determine convincingly whether such DNA corresponded to generalized transduction, or to remaining contamination. Among them, three contigs encoded ARG (according to the Resfinder results): two had a *tetR* gene on bacterial DNA, both coming from the dysbiotic VP7 piglet sample which was also one of the most contaminated samples, one had a *kanR* gene on a plasmid (Table 1).

Given the limited number of samples analyzed, few conclusions could be drawn concerning the differences between adult pigs and piglets, nor between animals treated with many/few antibiotics. We noted however that piglets had significantly higher abundances of *Caudovirales* compared to adult pigs, as well as fewer ssDNA eukaryotic viruses. The abundance of ARG genes found in microbiota did not correlate with the (recently applied) antibiotic treatments. The *tetR* genes were the most abundant overall, and most likely reflected the past practice, now forbidden in Europe, of antibiotic use for animal growth.

In conclusion, the consistent observations that ARGs are rarely encoded by phages is encouraging for the fight against antibiotic resistance, as well as for phage therapy. These results suggest that this type of mobile genetic element should not pose a threat, except in phages with high levels of generalized transduction. This is also good news in terms of safety for the use of phages to combat recalcitrant infections, as with the exception of those with high transduction levels, phages should not lead to ARG spread.

## Supplementary information

Supplementary information

Supplementary Table

Supplementary Table

Supplementary Table

Supplementary Table

## Data Availability

Raw sequencing data and SPAdes assembly outputs (>2 kb, contigs for VP samples, scaffolds for P samples, before the clustering step at 95% identity) are accessible through the NCBI, Bioproject PRJNA741980.
