# Supplementary material for: Analysis of viromes and microbiomes from pig fecal samples reveals that phages and prophages rarely carry antibiotic resistance genes
Source: ISME Commun. 2021 Oct 25;1:55. doi: 10.1038/s43705-021-00054-8 (PMC9723715; doi:10.1038/s43705-021-00054-8)
Supplement: Supplementary file 1 — Supplementary information [file 43705_2021_54_MOESM1_ESM.pdf]

Supplementary information

This file contains a Material and Method section, a section of Huge phage search, followed by the 4 supplementary figures, and finally the small supplementary tables 2, 4, 6 and 8.

1. Materials and Methods

Sampling

Fourteen farms in Brittany (France) were selected during year 2015, for pig feces sampling based on their various veterinary practices and more precisely, on their antibiotic treatment protocols. The antibiotic treatments for the fourteen animals used in our study are shown Suppl. Table 8. Since piglets are more heavily treated with antibiotics, five of the 14 samples were collected from piglets rather than adults.

Virome DNA sample preparation and sequencing

Viral particles were enriched from fecal samples following the route 5 protocol described in Castro-Mejia et al. [1] but with minor modifications. Two grams of frozen fecal sample were resuspended into 50 mL of PBS adjusted to 0.2 M NaCl, homogenized and agitated overnight at 4°C on a rolling device. After a prefiltration step on glass-fiber filters, samples were centrifuged at 5000 g 30 minutes at 4°C, and bacterial pellets were discarded. This centrifugation step was repeated until supernatants were clear (up to 3 times). Five grams of polyethylene glycol 8000 was then dissolved into the phage supernatants, and phages were left to precipitate overnight at 4°C. The precipitate was then centrifuged for 10 minutes at 15,000 g, after careful removal of PEG, and resuspended into 1 mL of SM buffer (50 mM Tris pH 7.5, 100 mM NaCl, 10 mM Mg-SO4). A iodixanol-SM step gradient was then formed in a 12-mL ultracentrifugation tube (Beckman, ultraclear tubes), by applying 6 mL of a solution at 20% iodixanol-SM, and then adding slowly with a Pasteur pipette, below this volume, 4.5 mL of a 45% iodixanol-SM solution. Finally, the phage sample (1.5 mL) was placed on top of the tube. The sample was ultracentrifuged for 5 h at 40 000 g, at 10°C. Most of the 14 gradients contained two visible

bands (see Figure A below). The lower band was the viral band, which was collected from the bottom of the tube and dialyzed against SM buffer (4 h) in two successive 1-L volumes (final volumes 3-5 ml after dialysis). Samples (0.9 mL) were then treated with DNAseI (2.5 U, 1h30 with Turbo DnaseI, Ambion) to remove residual free DNA.

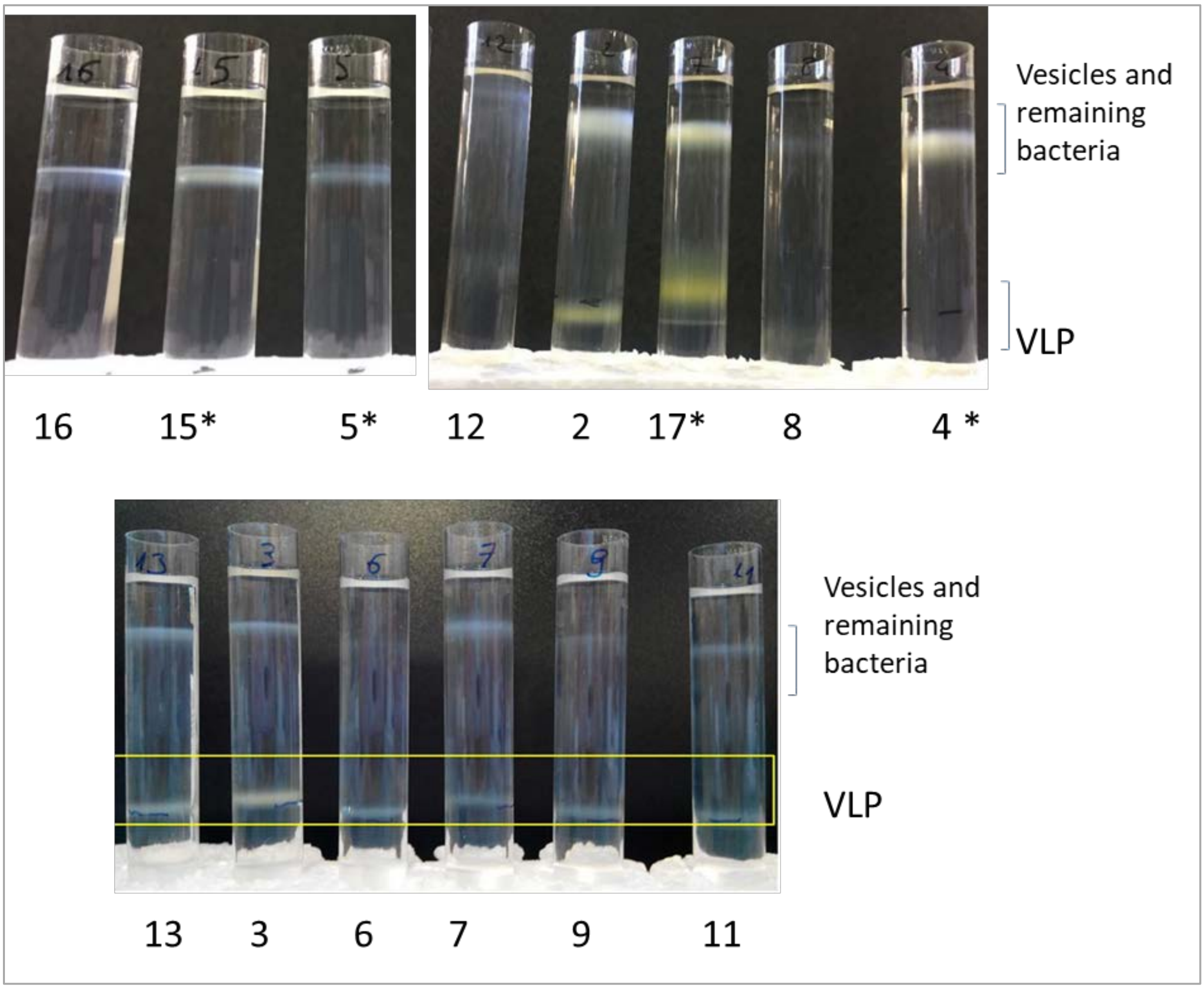

***Figure A**: Iodixanol gradients of the 14 pig samples (sample number is below each tube). A * indicates VLP samples that were filtered at 0,2µm, after gradient purification.*

To check for bacterial DNA contamination of the virome preparations prior to DNA extraction, qPCR was performed with universal 16S primers and compared to an *E. coli* total DNA standard. Total nanoparticle concentrations were also estimated with a Videodrop apparatus (Myriade), an apparatus that detects nanoparticles using light interferometry[2]. Most samples had less than 10 ng/mL of bacterial DNA for every 10^{10} nanoparticles and were not further treated prior to gradient loading. Filtration (0.2 µM pore size, polyethersulfone filters) was performed on four samples (VP4, VP5, VP15

and VP17) that were considered too heavily contaminated with bacteria. For viral DNA extraction, 1 mL of viral sample was added to a 1-mL solution of 50% phenol/50% chloroform-iso-amyl alcohol (24:1), manually mixed for 1 min, and centrifuged for 20 min at 12 000 g. The soluble phase was recovered and treated a second time with phenol-chloroform. The soluble phase was then added to an equal volume of chloroform-isoamyl alcohol (24:1), emulsified and centrifuged for 5 min at 12 000 g. DNA from the soluble phase was finally precipitated by adding sodium acetate (0.3 M), 1 µg of glycogen as the DNA carrier, and 0.6 volume of isopropanol. The DNA pellet was collected by centrifugation, washed with 70% ethanol, dried, and resuspended in 11 µL of 10 mM Tris pH 8. DNA was quantified using Qubit. Due to the insufficient total amount of DNA obtained (20 to 250 ng out of the 600 ng required), a multiple displacement amplification step (MDA, with kit Genomiphi2) was performed on 1 µL of each sample (90 min at 37°C), after microdialysis (0.02 µM VWPS filters, Millipore) to remove potential inhibitors. This step allowed for the inclusion of ssDNA viruses in the analysis. However, RNA viruses (which are the less abundant among known bacterial viruses) were excluded. Libraries were prepared using the Truseq kit and sequencing was performed on an Illumina Hiseq platform (2 x 150 nt, pair end), with an average depth of 50 million total reads per sample.

Virome read cleaning, assembly and contig treatments to constitute the VP reference dataset

Reads from each sample were trimmed using Trimmomatic[3] (ILLUMINACLIP:TruSeq3-PE.fa:2:30:10 LEADING:3 TRAILING:3 SLIDINGWINDOW:4:20 MINLEN:100), and dereplicated with the fastx-unique tool from the free version of the Usearch9 suite (R. Edgar, http://drive5.com/usearch). Reads were then paired again using the fastq_pair program (Edwards Lab), and unique reads (~30% of total) were kept as well. Assemblies were run separately on each sample using SPAdes[4] (version 3.13, parameters: --only-assembler -k 21,33,55,77,99,127) to include both paired and unique reads. The meta option in SPAdes, which does not permit inclusion of unique reads, was disregarded in order to keep as many reads as possible for assemblies, and improve contig lengths. A clustering step of viral contigs from all samples was performed at 95% nt identity using CDhit (psi-cd-hit.pl -c 0.95 -G 0 -g 1 -aS 0.9 -prog

blastn) and only contigs that were equal to or above 2 kb were retained for further analysis. The final viral dataset contained 7755 virome-de-porc (VP) contigs (out of the original 8090 contigs prior to clustering).

To examine the assembly completeness of the VP contigs dataset, dereplicated reads were mapped back on the 7755 contigs using the Bowtie2[5] with default parameters (allows multiple matches, with random affiliation). On average, 75% of total virome reads were mapped back on the VP collection. To generate abundance matrices, mapping result were treated with tools from the Samtool suite[6] (view, sort, index, idxstat), and the number of reads found on each contig was finally converted into reads per length of the contig (in kb) per million (RPKM). The VP matrix contained high RPKM values, ranging from 5 to 91 000.

Microbiota DNA sample preparation and sequencing

Total microbiota DNA extraction was performed according to protocol H of the international standards for human fecal samples (IHMS, http://microbiome-standards.org/index.php?id=Sop&num=007), starting from 0.2 g of frozen fecal sample. In short, purification steps involved guanidine thiocyanate/SDS sarkosyl treatment for 1h at 70°C, followed by bead beating to break open all bacteria, then repeated polyvinylpolypyrrolidone applications to remove polyphenols. DNA was then precipitated with isopropanol, washed and dried. This protocol should also break open the viral capsids, thanks to the initial guanidine thiocyanate protein denaturation step, although the question has never been specifically addressed, to our knowledge. However, the absence of any step to convert ssDNA into dsDNA prevented sequencing of ssDNA viruses. Libraries were prepared using the Truseq kit and sequencing was performed on an Illumina Hiseq platform (2x150 nt, pair end), with an average depth of 61 million total reads per sample.

Microbiota read cleaning, assembly and contig treatments to constitute the P reference dataset

The same steps applied for viromes were followed for total microbiota, with some simplifications. Briefly, reads of each sample were trimmed with Trimmomatic (same parameters as virome reads,

except MINLEN=125), and then directly assembled with SPAdes (same options as for viromes). A clustering step of of each individual microbial sample was performed at 95% nt identity with CDhit, and only contigs of a size equal to or above 2 kb were retained for further analysis. The microbial dataset comprised 220,000 pig (P) contigs (from 271,163 contigs prior clustering).

To examine the assembly completeness, reads were mapped back onto the P contig dataset using Bowtie2 on default parameters. On average, 69% of microbial reads were mapped back on the P contigs collection. To generate abundance matrices, mapping results were treated with tools from the Samtools suite, and the number of reads found on each contig was finally converted into RPKM. The P contigs matrix contained RPKM values that were 10-times lower than the VP matrix, ranging from 0.71 to 6890.

To estimate the main phyla present in the microbiome samples, a taxonomic affiliation was determined using Kaiju [7] for the top 10 585 P contigs with a global abundance above 10 RPKM. To sum up contig abundances by phyla, those which might belong to the same microbial species were binned into clusters based on kmer composition and co-occurrence with VAMB [8]. A single contig per VAMB cluster was conserved in the abundance matrix, which ended up containing 5862 contigs (~species). Moreover, in the context of ARG analyses, Kaiju-based taxonomic assignment of interesting contigs was complemented with a BLASTn against the nt database (retaining the best hit with E-value below 10^{-50}, when present).

Bacterial contamination level of the viral-enriched DNA

The amount of bacterial DNA contamination of the virome reads was estimated using two methods. First, reads were mapped onto the 16S SILVA collection[9] with Bowtie2. All samples had a ratio of 16S matching reads over total reads (prior dereplication) of 2×10^{-4}, which is a cut-off for viromes with satisfactory-level purity[10]. The maximal 16S read ratio was 3×10^{-5} in sample VP2 (see Suppl. Table 2). Second, reads were submitted to the viromeQC program[11], which estimates a viral enrichment yield. This is done by comparing the frequency of reads matching with 16S, 23S and ribosomal protein

encoding genes, to that expected for global microbial metagenomics samples. Enrichment was 100-fold, except for 3 samples (lowest value 7-fold, sample VP2, Suppl. Table 2). ViromeQC results matched well with the 16S method, and showed that the pig viromes were essentially viral in content.

Contig annotations and ARG search

VP and P contigs were annotated using Prokka [12] (version 1.13 with --addgenes and –rnamer options). Following annotation, three ARG searches were compared for the VP contigs: (i) hmmscan against ResFam profiles ([13] –cut_ga option), as in our previous report [14]; (ii) BLASTp against the MUSTARD database [15] (≥ 95% id and ≥ 90% alignment cut-offs), built in part with ARG genes found in the human gut; (iii) BLASTn against the version 4.0 release of Resfinder [16], with parameters ≥ 90% identity, ≥ 60% coverage. For microbial P contigs, only the Resfam and Resfinder searches were performed. A deeper analysis was then conducted on the most prevalent contigs. BLASTn search against nt (as of November 2020) with contig P4_NODE_19877 (2256 bp, contains a *tetR* gene), indicated that among the top 176 BLASTn hits (≥ 99% identity, 100% coverage), 41 were *Clostridiales*, and 11 *Bifidobacteriaceae* complete genomes. In the vicinity of the *tet* gene, genes annotated as *traG*, *mobC* and relaxase were found, suggesting the presence of an ICE.

Elements of VP contig classification

-single-stranded (ss) DNA viruses: A total of 2807 ssDNA viruses were recognized. *Petitvirales* were recognized either because they were specifically mentioned ("non-*Caudovirales* gene enrichment" score) in the Virsorter output, or by searching for terms 'VP1', 'VP4', and 'Pilot' in the annotated contigs of the VIBRANT output. The ssDNA eukaryotic viruses were mostly detected thanks to Megablast searches against the viral section of nt, and therefore directly affiliated. A 'REP_TLCV' (rep gene of tomato-leaf curl virus) annotation in VIBRANT files allowed to detect some additional eukaryotic viruses. *Tubulavirales* were detected thanks to Inovirus-detector [17]. Most (272 of 344 total inoviruses) were circular, and these were analyzed into more details. A vCONTACT2 clustering of these 252 circular contigs revealed 50 clusters with a quality above 0.5, involving altogether 65% (179 phages) of all input

genomes. Pairwise ANI (average nt identity) were also computed for all genomes sharing the same *gpI* gene, a gene typical of filamentous phages and needed for virion morphogenesis. Species membership was defined for pairs with ANI above 0.95, and genus boundary ANI > 0.75. A summary of the 272 circular genome properties is listed in Suppl. Table 3.

-double-stranded (ds) DNA viruses: All viral contigs not recognized as ssDNA viruses were placed abruptly into the *Caudovirales* category (2999 in total). These were clustered with vCONTACT2 [18], together with the 2191 reference phages from Viral Refseq v88, and 23 additional intestinal phages such as LAK phages[19], phages infecting *Faecalibacterium prausnitzii*[20] and *Roseburia intestinalis*[21]. For 1675 of the 2999 VP contigs, a cluster was formed (478 clusters altogether), but only 18 of them contained reference phage genomes. All clusters of a quality above 0.5 (as indicated in the 'quality' column of the clusters output file of vCONTACT2) were considered further, and the top 7 are listed in Suppl. Table 4. These vCONTACT2 clusters were used to coalesce the most abundant *Caudovirales* contigs.

Phage genome annotations were performed by running a BLASTp of ORFs predicted with Prokka against the NR database at NCBI (E-val < 10^{-5}), and if needed HHpred against PDB (P > 98%) using the online Tübingen Toolkit Webserver[22], and alignments maps were done with Easyfig[23].

Partitioning active phage versus inactive prophage contigs in the microbial P dataset, among contigs detected as viral with VIBRANT.

A schematic summarizing the classification of active phage and inactive prophage contigs is shown in Suppl. Figure 2a. Among the 220 000 P contigs, the viral ones were searched with VIBRANT. This gave 16 612 positive contigs. To complement this collection, 328 contigs matching by BLASTn to a VP contig (> 95% nt identity) or affiliated by Kaiju as viral, were added (total 16 940 viral P contigs). We reasoned that this set of viral contigs represented two very different situations, either virulent or temperate phages replicating and forming virions (category "active phage"), or dormant prophages that do not manifest viral activity (ie no capacity to form a free virion, at least in the ecosystem studied, category

“prophage”). We took advantage of the virome reads to partition this set into either “active phage” if the viral P contig was matched by virome reads, or “prophage” if not, as described below.

We were also interested in the overlap between VP contigs and these viral P contigs. In a first step, therefore, the virome VP contigs were placed together with microbiome viral P contigs and dereplicated (95% identity, 90% coverage), generating a set of 23 181 non redundant viral contigs. Among them, 6658 were found only in the VP set (54 Mb cumulative length). There were 660 contigs in the “overlap” category, where contigs are representing both viral P and VP segments, with in some cases many small P contigs matching a large VP contig, and also the reverse situation, indicating that viral assembly was incomplete in both cases, unsurprisingly (24 Mb). The last 15 873 contigs were found only in the P assembly (178 Mb).

In a second step, virome reads were mapped onto the complete collection of 23 181 non redundant viral contigs (Suppl. Figure 2b). We reasoned that the modest overlap of the VP contigs with P ones (only 14% of VP have a match to P) could be the result of partial assembly, so that parts of the same phage may be assembled in each set. Therefore, it was expected that some of the P contigs having no overlap with any VP contig might still be corresponding to active phages. In this mapping, the VP contig with lowest cumulative abundance across the 14 samples had a RPKM value of 2.6. We then considered as “active viral contigs” all P contigs with global RPKM abundance above 2.6. This added 3049 P contigs as active viral contigs (39 Mb). In total, of the 16 940 starting viral P set, 4021 contigs (23.7%) belonged to the active phage category (corresponding to these 3049, together with 972 P contigs present or included into VP contigs of the “overlap” set), while the rest was considered inactive prophages.

Finally, to confirm the partial assembly hypothesis, the reciprocal mapping of microbiota P reads onto the same contig dataset was performed (Suppl. Figure 2c). In this mapping, the viral-P contig with lowest abundance across the 14 samples had a RPKM value of 0.45, so that all VP contigs above this value were counted as covered. The matrix revealed indeed that 3264 contigs of the VP set,

representing 20% of the total RPKM of this matrix, were covered by P reads. Inspection of the remaining, not-covered VP contigs (3394) revealed that 70% were ssDNA viruses.

Host predictions for interesting phage clades

For the 7755 VP contigs, host prediction was first attempted by searching for matching CRISPR spacers, using the recent spacer database from Dion et al.[24] and the default parameters (a maximum of two mismatches allowed). A host was predicted for 469 of these contigs (6% of total contigs). Next, a set of spacers was extracted from our P contigs (of all sizes, including those below 2 kb). For these spacers, CRISPR arrays were first identified using CRISPRDetect v2.2[25]. Spacers were then extracted from the CRISPRDetect GFF output file along with the following metadata: contig ID, start and end positions on the contig, length, sequence, orientation and position inside the locus. Spacers were assigned a unique ID and a custom BLAST database was generated (n = 14 941 spacers). Next, BLASTn v2.9.0 was used to perform pairwise alignments between the P contig spacers and the selected viral contigs, applying the same filters as previously described[24]. A total of 1075 (14%) VP contigs matched the in-house spacer collection. For 148 of the P contigs containing such matching spacers, a taxonomical affiliation could be proposed, based on the Kaiju indication followed by a BLASTn search (E-value < 10^{-16}) against the Bacteria section of the nt database. As expected, affiliation was more successful using the in-house spacer collection, although in most cases, taxonomical affiliation of these in-house P contigs was unsuccessful (many very short contigs).

For the two clades of phages mostly focused in our study, 272 inoviruses and 94 Oengus-like phages, 15 and three hosts were predicted by this first method, respectively. Three additional tests were performed on this subset: (1) submission to the spacer collection at JGI (https://img.jgi.doe.gov/cgi-bin/vr/main.cgi) and applying the two-mismatch cutoff as above, (2) BLASTn search against NCBI nt (E-value < 10^{-4} and coverage > 10%), and (3) WiSH[26]. WiSH computes conditional probabilities of all 9-mers in bacterial genomes of the database (2155 bacterial genomes meaningful for the ecosystem were taken) and compares them to that of the query phage. The first output is the probability

(expressed as log-likelihood) that the given phage will infect each of the bacteria in this collection. The closer the log-likelihood is to 0, the more likely the host is correct. A log-likelihood is also calculated for the phages that do not infect the bacteria of this same collection. By comparing these two log-likelihoods, a second statistic (a p-value) gives the confidence of the WiSH prediction. Four inoviruses and four phages of the Oengus cluster with known hosts were tested as controls. Only the five best log-likelihood with a p-value inferior or equal to 0.05 for each phage contig were considered as putative hosts. Next, a filter was applied on the log-likelihood values, which had to be below those obtained for the control phages. Finally, the bacteriophages with 4 to 5 predictions from the same taxonomic order were considered sure and kept.

2. Huge phage search

We attempted to detect any so-called "huge phage" (size > 200 kb)[27] among virome VP contigs. VP homologs to the reported 361 huge phage genomes[27] were searched by a simple BLASTn comparison (E-val < 10^{-5}) to the huge phages. Very few hits were found (30) and none with a query coverage above 14%. Then a more sensitive and more restricted search was done with tBLASTx on a subset of large virome VP contigs and microbiota P contigs. These included the 21 VP contigs of a size above 150 kb, a 381 kb P contig considered viral due to its homology to a VP contig by BLASTn, and 5 VAMB bins having both a Kaiju annotation as viral and a cumulated size above 180 kb. These contigs were compared to the 35 closed_circular_curated huge phages[27] and the 15 complete *Prevotella* megaphages[19] with cutoffs placed at 30% query cover, 65% (amino-acid) identity and E-value 10^{-3}. Four distant relatives of complete phage AOT2015-SM01_PERU_trim_clean_scaffold_10 (PERU for short, 201 kb) were found. For the first of these, a long contig was present both in the microbiome and virome set. In the microbiome, this contig (P12_NODE_16) was 183 kb long, and its central section matched perfectly with the virome contig VP12_NODE3 (123 kb), confirming its viral nature. The three additional contigs (175-154 kb) came from three viromes VP2, VP9 and VP12. A

Clostridium host was predicted for one of them (VP2_NODE1) because of a CRISPR spacer[24]. The largest of these 4 contigs was manually annotated, and all 4 were aligned with PERU using tBLASTx and Easyfig (see figure A below).

A fraction of the structural module (head genes) and the whole replication module genes were shared between these phages. Interestingly, P12_NODE_16 had long tail genes (absent from PERU and the 3 other contigs) and no detectable sheath encoding gene, suggesting that it is a siphovirus. A few ribosomal genes and eukaryote-like ribonucleoprotein genes (major vault-like protein-encoding genes) were also predicted.

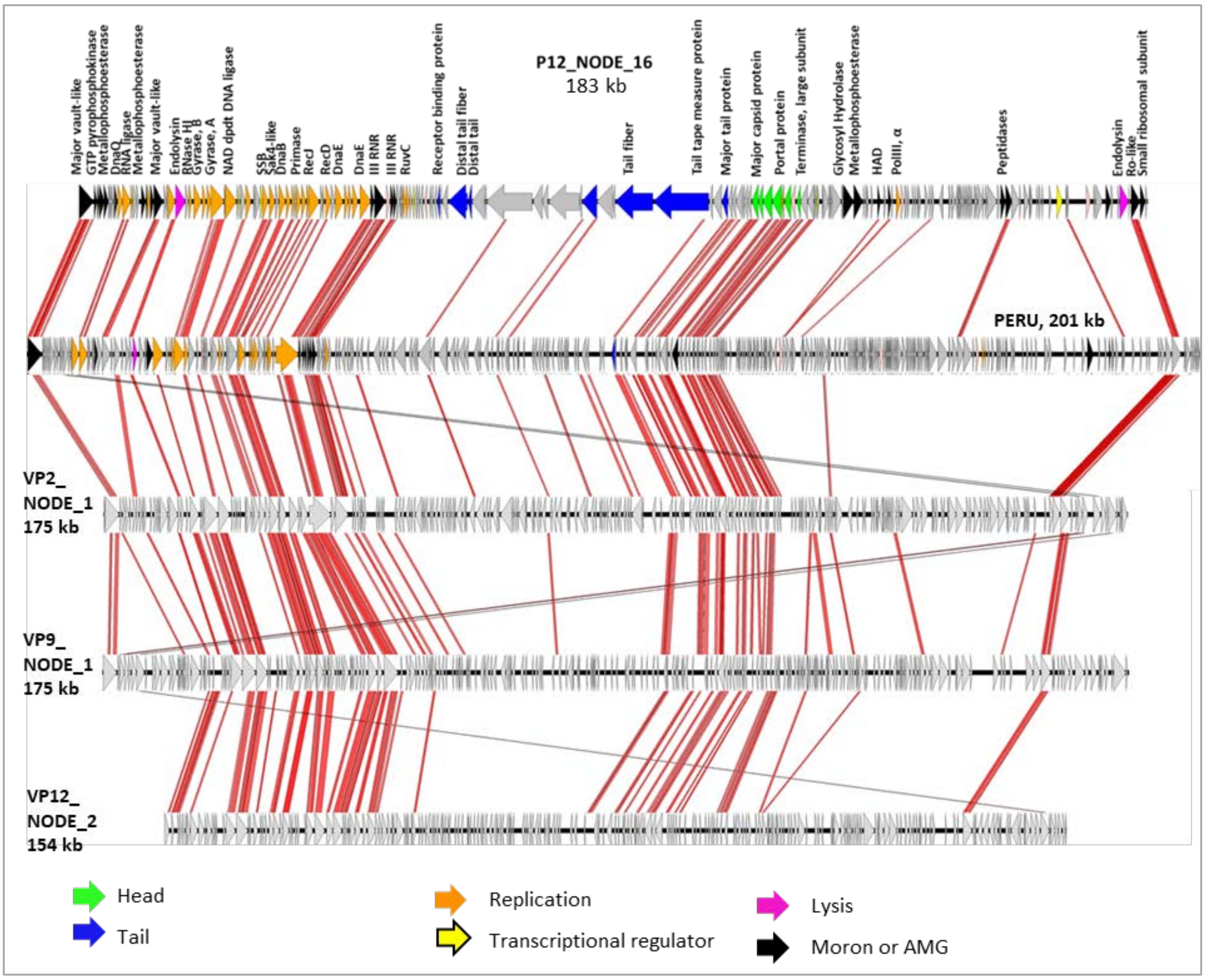

Figure B. *Pairwise comparisons between a pig microbiota contig, AOT2015-SM02_PERU (201 kb), and 3 pig virome contigs by tBLASTx, (minimal id. 30%, minimal length 100 bp) using Easyfig. +1 positions were translated on the pig contigs, to fit PERU starting position. P12_NODE6 was annotated manually. Gene abbreviations: DnaQ, DnaQ exonuclease; SSB, single- stranded DNA binding protein; Sak4, Sak4-like recombinase; DnaB, DnaB helicase; RecJ, RecJ single-stranded DNA specific exonuclease; RecD, RecD helicase; DnaE, DnaE DNA polymerase III epsilon subunit; III RNR, Class III ribonucleotide reductase; RuvC, RuvC crossover junction endodeoxyribonuclease; HAD, haloacid dehalogenase-like hydrolase, PolIII α, DNA polymerase III alpha subunit; AMG, auxiliary metabolic gene.*

1. Castro-Mejia, J.L. *et al.* Optimizing protocols for extraction of bacteriophages prior to metagenomic analyses of phage communities in the human gut. *Microbiome* **3**, 64 (2015).
2. Boccara, M. *et al.* Full-field interferometry for counting and differentiating aquatic biotic nanoparticles: from laboratory to Tara Oceans. *Biomed Opt Express* **7**, 3736-3746 (2016).
3. Bolger, A.M., Lohse, M. & Usadel, B. Trimmomatic: a flexible trimmer for Illumina sequence data. *Bioinformatics* **30**, 2114-20 (2014).
4. Bankevich, A. *et al.* SPAdes: a new genome assembly algorithm and its applications to single-cell sequencing. *J Comput Biol* **19**, 455-77 (2012).
5. Langmead, B. & Salzberg, S.L. Fast gapped-read alignment with Bowtie 2. *Nat Methods* **9**, 357-9 (2012).
6. Li, H. *et al.* The Sequence Alignment/Map format and SAMtools. *Bioinformatics* **25**, 2078-9 (2009).
7. Menzel, P., Ng, K.L. & Krogh, A. Fast and sensitive taxonomic classification for metagenomics with Kaiju. *Nat Commun* **7**, 11257 (2016).
8. Nissen, J.N. *et al.* Improved metagenome binning and assembly using deep variational autoencoders. *Nature Biotechnology* (2021).
9. Quast, C. *et al.* The SILVA ribosomal RNA gene database project: improved data processing and web-based tools. *Nucleic Acids Res* **41**, D590-6 (2013).
10. Roux, S., Krupovic, M., Debroas, D., Forterre, P. & Enault, F. Assessment of viral community functional potential from viral metagenomes may be hampered by contamination with cellular sequences. *Open Biol* **3**, 130160 (2013).
11. Zolfo, M. *et al.* Detecting contamination in viromes using ViromeQC. *Nat Biotechnol* **37**, 1408-1412 (2019).
12. Seemann, T. Prokka: rapid prokaryotic genome annotation. *Bioinformatics* **30**, 2068-9 (2014).
13. Gibson, M.K., Forsberg, K.J. & Dantas, G. Improved annotation of antibiotic resistance determinants reveals microbial resistomes cluster by ecology. *ISME J* **9**, 207-16 (2015).
14. Enault, F. *et al.* Phages rarely encode antibiotic resistance genes: a cautionary tale for virome analyses. *ISME J* **11**, 237-247 (2017).
15. Ruppe, E. *et al.* Prediction of the intestinal resistome by a three-dimensional structure-based method. *Nat Microbiol* **4**, 112-123 (2019).
16. Bortolaia, V. *et al.* ResFinder 4.0 for predictions of phenotypes from genotypes. *J Antimicrob Chemother* **75**, 3491-3500 (2020).
17. Roux, S. *et al.* Cryptic inoviruses revealed as pervasive in bacteria and archaea across Earth's biomes. *Nat Microbiol* **4**, 1895-1906 (2019).
18. Bin Jang, H. *et al.* Taxonomic assignment of uncultivated prokaryotic virus genomes is enabled by gene-sharing networks. *Nat Biotechnol* **37**, 632-639 (2019).
19. Devoto, A.E. *et al.* Megaphages infect Prevotella and variants are widespread in gut microbiomes. *Nat Microbiol* **4**, 693-700 (2019).
20. Cornuault, J.K. *et al.* Phages infecting Faecalibacterium prausnitzii belong to novel viral genera that help to decipher intestinal viromes. *Microbiome* **6**, 65 (2018).
21. Cornuault, J.K. *et al.* The enemy from within: a prophage of Roseburia intestinalis systematically turns lytic in the mouse gut, driving bacterial adaptation by CRISPR spacer acquisition. *ISME J* **14**, 771-787 (2020).
22. Gabler, F. *et al.* Protein Sequence Analysis Using the MPI Bioinformatics Toolkit. *Curr Protoc Bioinformatics* **72**, e108 (2020).
23. Sullivan, M.J., Petty, N.K. & Beatson, S.A. Easyfig: a genome comparison visualizer. *Bioinformatics* **27**, 1009-10 (2011).
24. Dion, M.B. *et al.* Streamlining CRISPR spacer-based bacterial host predictions to decipher the viral dark matter. *Nucleic Acids Res* **49**, 3127-3138 (2021).
25. Biswas, A., Staals, R.H., Morales, S.E., Fineran, P.C. & Brown, C.M. CRISPRDetect: A flexible algorithm to define CRISPR arrays. *BMC Genomics* **17**, 356 (2016).

26. Galiez, C., Siebert, M., Enault, F., Vincent, J. & Soding, J. WIsH: who is the host? Predicting prokaryotic hosts from metagenomic phage contigs. *Bioinformatics* **33**, 3113-3114 (2017).
27. Al-Shayeb, B. *et al.* Clades of huge phages from across Earth's ecosystems. *Nature* **578**, 425-431 (2020).

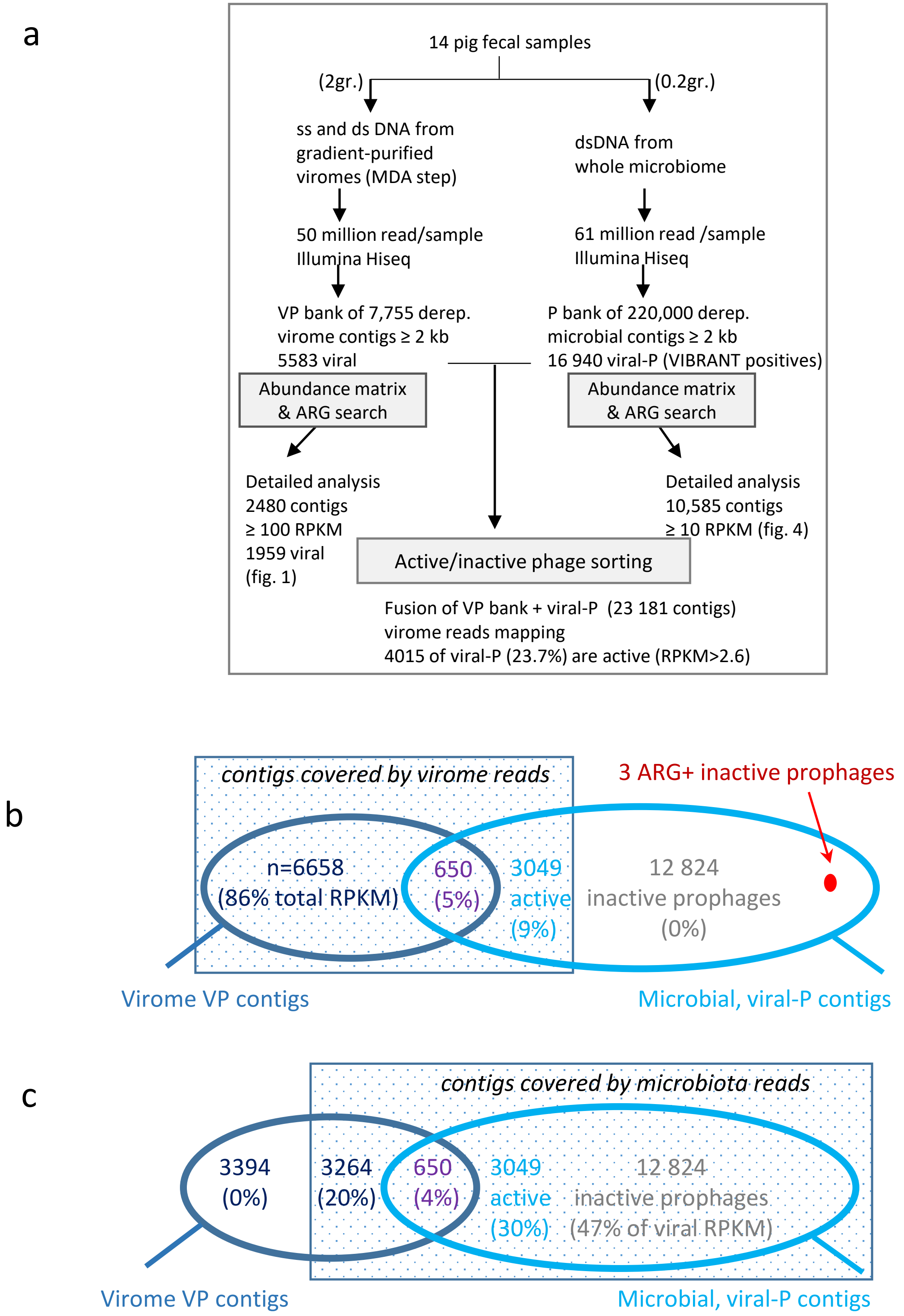

Supplementary Figure 1. **a**: Method for detection of viral contigs in microbiomes and partition between active and inactive ones. **b** (taken from Figure 5, for comparison with panel c): Representation of the 23 181 contigs issued either from virome assembly (VP) or the viral subset of the microbiota assembly (viral-P), with their small overlap of 650 common contigs. Mapping VP reads to this dataset reveals a large fraction of inactive prophages among microbiota viral-p contigs, as well as 3049 active viral-P contigs, representing 9% of the total RPKM. C. The reciprocal, microbial reads mapping on the same set of 23 181 contigs reveals microbial reads mapping to 3264 VP contigs, representing 20% of the viral-RPKM signal.

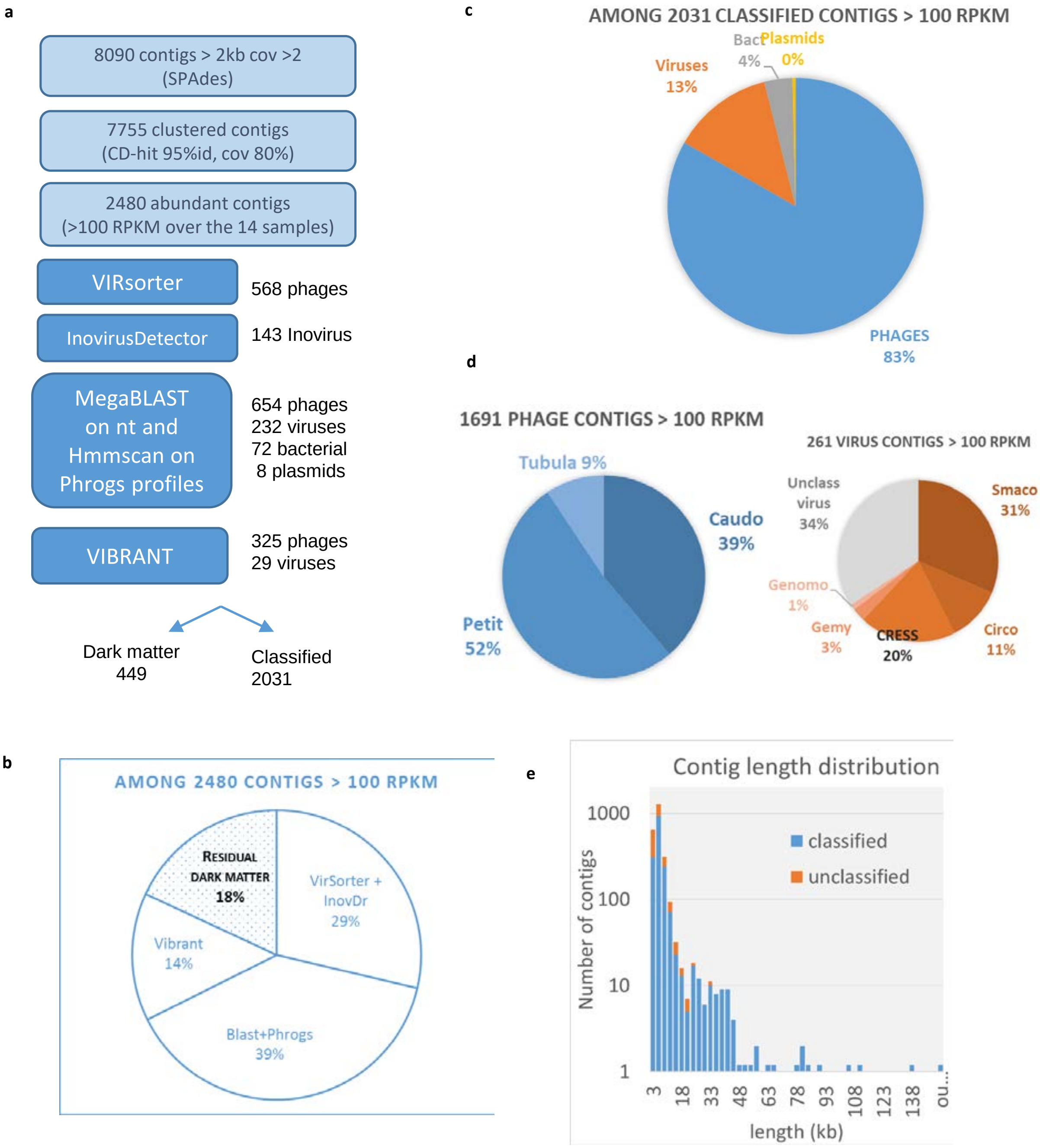

Supplementary Figure 2: Classification of 2480 VP contig with high abundance and size > 2kb. A. Strategy to affiliate contigs. B. 82 % of contigs >2kb are affiliated. C. Most recognized contigs are phages. D. Main categories of phages and viruses. Inoviridae constitute 9 % of all phage contigs, and Smacoviruses is the most populated category of viruses in pig viromes. E. Size distribution of contigs, and proportion of unclassified in each size class.

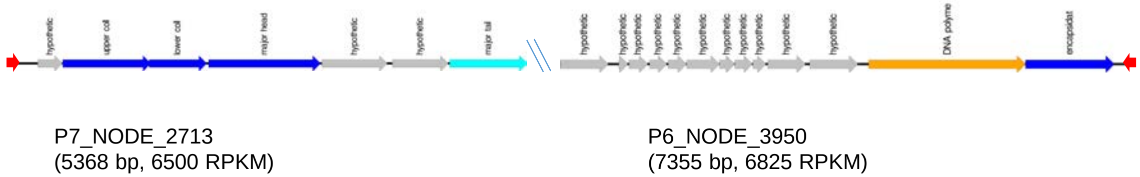

Supplementary Figure 3 The genetic map of the dominant phage in sample P7 reveals a typical Picovirinae organisation. Contigs were annotated with Hhpred, 259 bp terminal inverted repeats are shown in red. The P7_NODE_2713 contig was recognized as viral by VIBRANT, unlike the othercontig.

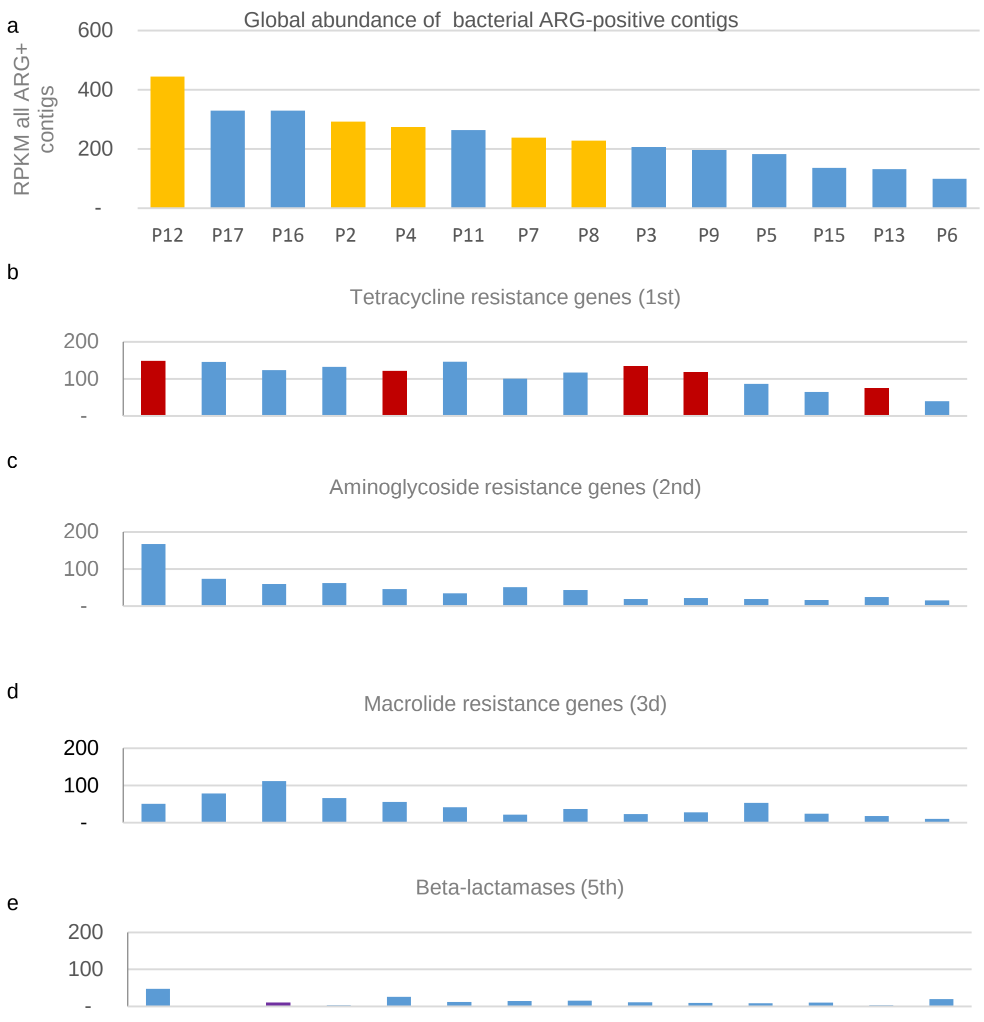

Supplementary Figure 4. **a**. Global abundance of ARG-positive contigs in the 14 pig fecal sample microbiota. Piglet samples are shown in yellow, adults in blue. **b to e**, in the same order as in A, abundance of the main categories of antibiotic resistance genes (rank of abundance. Red bars: animals treated with tetracycline. Purple bar: animal treated with beta-lactams.

Supplementary Table 2: bacterial DNA contamination of virome samples

Sample	Filtered	Total reads	Reads mapped on 16S (Silva)	Freq 16S reads	viromeQC enrichment
VP2		45 413 231	1287	2.83E-05	7
VP3		38 432 018	23	5.98E-07	100
VP4	YES	39 665 645	68	1.71E-06	100
VP5	YES	44 131 406	2	4.53E-08	100
VP6		41 894 101	44	1.05E-06	100
VP7		43 350 198	769	1.77E-05	26
VP8		48 854 252	281	5.75E-06	62
VP9		46 636 927	171	3.67E-06	100
VP11		37 192 968	104	2.80E-06	100
VP12		43 095 618	63	1.46E-06	100
VP13		40 804 259	104	2.55E-06	100
VP15	YES	39 981 040	4	1.00E-07	100
VP16		43 004 890	19	4.42E-07	100
VP17	YES	43 630 846	11	2.52E-07	100

Supplementary Table 4: vCONTACT2 VP clusters with largest numbers of contigs

Name	N VP contigs	ref phage	av size complete genome
cluster_1	94	Faecalibacterium phage Oengus	58 kb
cluster_23	38	None	38 kb
cluster_62	17	None	125 kb
cluster_99	17	Lactococcus phage r1t	33 kb
cluster_100	16	None	29 kb
cluster_88	8	Bacteroides phage B40-8	46 kb
cluster_328	7	Bacteroides Crassphage01	90 kb

Supplementary Table 6: ARG detection in microbiota, global statistics

Sample	animal type	N ORF	Mb, all contigs	N ARG ResFam	N ARG Resfinder	ARG ResFam /N ORF	ARG ResFam /Mb	ARG Resfinder /N ORF	ARG Resfinder /Mb
P3	adult	98 507	97.868	102	19	1.04E-03	1.04	1.93E-04	0.19
P5	adult	128 655	132.811	89	26	6.92E-04	0.67	2.02E-04	0.20
P6	adult	154 692	148.622	101	19	6.53E-04	0.68	1.23E-04	0.13
P9	adult	143 904	141.983	98	21	6.81E-04	0.69	1.46E-04	0.15
P11	adult	125 978	127.457	175	26	1.39E-03	1.37	2.06E-04	0.20
P13	adult	121 095	117.966	86	19	7.10E-04	0.73	1.57E-04	0.16
P15	adult	124 547	120.031	100	19	8.03E-04	0.83	1.53E-04	0.16
P16	adult	112 647	114.048	161	31	1.43E-03	1.41	2.75E-04	0.27
P17	adult	77 636	76.678	122	16	1.57E-03	1.59	2.06E-04	0.21
P2	piglet	91 912	92.012	131	11	1.43E-03	1.42	1.20E-04	0.12
P4	piglet	144 329	144.556	183	20	1.27E-03	1.27	1.39E-04	0.14
P7	piglet	72 106	71.658	106	20	1.47E-03	1.48	2.77E-04	0.28
P8	piglet	117 983	118.835	143	11	1.21E-03	1.20	9.32E-05	0.09
P12	piglet	111 326	113.541	167	21	1.50E-03	1.47	1.89E-04	0.18
sum		1 087 661	1 077.46	1034	196				
average						1.13E-03	1.13	1.77E-04	0.18
std dev						3.54E-04	0.35	5.49E-05	0.05

Supplementary Table 8: pig samples, and antibiotic treatments

sample #	Animal type	Antibiotic treatments on animal sampled
3	adult, female	oxytretracycline
5	adult, female	
6	adult, female	
9	adult, female	oxytretracycline
11	adult, female	dexalone, marbofloxacine
13	adult, female	doxycycline
15	adult, female	
16	adult, female	TMP, sulfadiazin, tylosin, amoxicillin, marbofloxacin
17	adult, female	
2	piglet	millicoli, tiamvet
4	piglet	oxytetracycline
7	piglet	
8	piglet	lyncomycin, colistine
12	piglet	oxytetracycline
